# Impact of Ethical Leadership on Employee Engagement: Role of Self-Efficacy and Organizational Commitment

**DOI:** 10.3390/ejihpe11030071

**Published:** 2021-08-25

**Authors:** Fouzia Ashfaq, Ghulam Abid, Sehrish Ilyas

**Affiliations:** 1 Department of Management Sciences, Lahore College for Women University, Jail Road, Lahore 54000, Pakistan; sehrish.ilyas@lcwu.edu.pk; 2 Department of Business Administration, Kinnaird College for Women, 93 Jail Rd, G.O.R. - I, Lahore 54000, Pakistan; ghulam.abid@kinnaird.edu.pk

**Keywords:** employee engagement, ethical leadership, organizational commitment, self-efficacy

## Abstract

The aim of this study was to examine the roles of self-efficacy and organizational commitment in the sequential mediation of the relationship between ethical leadership and employee engagement. Data were collected through self-reported questionnaires of employees from private and public sector organizations of Pakistan. We opted for a three-wave time-lagged design, and we used the PROCESS macro by Hayes on a sample of 211 employees (35% male, 65% female) via the 2000 re-sample bias-corrected bootstrap method. The results show a significant relationship between ethical leadership and employee engagement with mediating effects of self-efficacy and organizational commitment. Self-efficacy and organizational commitment fully mediated the relationship. The results provide insight into the understanding of employee behavior, particularly in the presence of moral leadership. Drawing on the conservation of resource theory, we examined how ethical leader support enables employees to invest their resources into positive outcomes. Theoretical and practical implications are discussed.

## 1. Introduction

An ethical style of leading is essential in stimulating ethical conduct in the workplace. While transmitting the ethical values of the institution [1], ethical leadership cultivates employee commitment to the organization [2]. On this topic, empirical research is at a relatively emerging stage [3]. In terms of establishing ethical norms, it is presumed that ethical leadership plays an important role [4]. Ethical leadership is defined as “the demonstration of normatively appropriate conduct through personal actions and interpersonal relationships, and the promotion of such conduct to followers through two-way communication, reinforcement, and decision making” [4], p. 120. The clarity that ethical leaders maintain in their expectations, communication, and responsibilities is reciprocated with a more committed and engaged workforce within the organization [5]. A growing number of studies have supported the significance of ethical leadership and its positive effects on the behavioral outcomes of followers [6,7,8].

Drawing on Albert Bandura’s social cognitive theory [9], self-efficacy initiates personal achievements as well as the motivation and well-being of employees. The theory is based on peoples’ beliefs rather than what is objectively true [9]. Organizational scholars treat it as a motivational trait [10]. According to Chen et al. [11] and Shelton [12], the integration of success and failures of an individual’s experiences become a source of confidence in them. Self-efficacy has the potential to foster one’s behavior in erratic conditions by changing individuals’ expectations [13]. Employees with high self-efficacy become resilient, have confidence that they can meet challenges, and turn out to be more committed and engaged in their work [14].

According to Hunt et al. [15], organizational commitment defines employees’ interest in and connection to an organization. Committed employees identify themselves with the goals and objectives of their organizations and strive for their association with the organization to continue their membership [15]. The research also reveals that organizations, as well as individuals, gain benefits from the high commitment of the employees, and both experience adverse effects of low commitment [16]. Organizational commitment is associated with the increased engagement of the employees and their satisfaction [17].

Contemporary studies are now focusing on employee engagement [18], which is described as a valuable behavior of employees that shows their commitment [19]. Significant efforts have been made by numerous researchers to establish the alignment between employee engagement and leadership styles [20]. Recent findings argue the role of an ethical style of leadership on the performance of employees [21,22]. However, enough attention has still not been given to the intervening mechanism that exists between ethical leadership and employee engagement. According to Carasco-Saul et al. [23], the gap that exists in understanding which leadership style changes employee behavior, in terms of engagement, needs to be bridged. Moreover, little research exists on the direct link between ethical leaders’ attributes and followers’ engagement at the workplace, as well as on the underlying mechanism that facilitates this link [1,24].

This study was grounded in the prediction of the conservation of resource theory [25] to address the above-mentioned existing research gaps. The conservation of resource theory proposes that people invest resources to attract further, more valuable resources. This investment of resources allows them to attain goals set earlier [25]. The conservation of resource theory considers a leader’s positive style as a great organizational resource that increases employees’ energy levels and their efficacy [26]. According to Eldor and Harpaz [27], the resources of the organization may be regarded as the main antecedent that drives work engagement in employees. In this vein, employees who possess increased levels of engagement at the workplace have a tendency to invest their possessed resources to achieve outcomes that satisfy them, that is, commitment and engagement. Hence, we may expect that leaders may increase employee engagement by increasing their efficacy through being supportive resources for employees.

The current study aims to investigate why ethical leadership may assist employees in becoming engaged in the workplace. Furthermore, we investigated how ethical leadership supports employees to become more engaged and examined the underlying roles of self-efficacy and organizational commitment.

## 2. Theoretical Underpinning

The current research is supported by the conservation of resource theory. According to a less examined component of the theory, resources in the workplace are invested to build new resources [25]. Support from leadership offers valuable resources in the form of physical and emotional resources for their employees. That is, the employee who receives such resources develops more resources from their available resources, as per the expectations. Hence, a positive environment shaped by leadership creates self-efficacy and organizational commitment. According to Zhou, Wang, Chen, and Shi [28], leaders’ conduct, like resources, has a significant impact. Employees reciprocate supportive leadership by investing this obtained resource to achieve greater engagement in their work.

### 2.1. Ethical Leadership and Self-Efficacy

The growing attention ethical leadership has been attracting is attributed to its capacity to tap employees’ positive attitudes towards everyday assignments at the workplace [29]. The extent of influence that leadership creates on the attitudes and behaviors of employees is not limited to the managerial aspect; nevertheless, it also has ethical implications [30]. Dirks and Ferrin [31] consider the stature of a leader as the bearing of a powerful position that can affect employees’ work attitudes and behavior in mainly, but not limited to, two domains. First, the ethical attitude of a leader who is honest, credible, and gives autonomy and opportunities to workers makes employees feel indebted to reciprocate the respect, care, and support with positive attitudes related to the job. Second, fairness in job evaluations, performance, and promotions creates optimism and commitment in employees [32], making them more efficient.

In line with the above reasoning, for the current study, we predicted that leaders’ ethical conduct is positively related to the self-efficacy of employees.

**Hypothesis** **1** **(H1).** *Ethical leadership is positively related to employees’ self-efficacy.*

### 2.2. Self-Efficacy and Employee Commitment

The proposed model suggests that important influences on commitment can be found in three general areas of organizational life. A major advantage of the present study is that it allows for the simultaneous examination of the various antecedents in order to identify the relative strength of each relationship with commitment. Previous studies were more focused and typically did not provide an adequate test of the relative weights of each antecedent [33]. Furthermore, the meta-analysis substantiates that self-efficacy is strongly related to commitment [34]. Hence, the current study proposes a positive relationship between self-efficacy and employees’ commitment.

**Hypothesis** **2** **(H2).** *Self-efficacy is positively associated with employees’ commitment to the organization.*

### 2.3. Employee Commitment and Employee Engagement

Employee engagement is believed to include employee organizational commitment, due to which, employees form longstanding relationships with their workplace [19]. Engagement and energy for work along with the work environment lead to employees’ commitment [35]. Macey et al. [36] contended that motivated employees, when given autonomy, become highly engaged with their work. Furthermore, in addition to having social influence, leadership also plays a constructive role in enhancing engagement in employees [37].

According to Steers [38], committed employees have a strong desire to remain associated with the organization. He further argued that their commitment elicits positivity from them, and it creates a strong drive to work and contribute to goal attainment. Therefore, the commitment presumes that committed employees would expend greater efforts on their job and encounter more engagement [38]. Hence, we expect:

**Hypothesis** **3** **(H3).** *There exists a positive relationship between employees’ commitment to the organization and employees’ engagement.*

### 2.4. Self-Efficacy as a Mediator between Ethical Leadership and Employee Engagement

Bandura [39] argued that self-efficacy exerts a significant influence on employees’ choices, efforts, and consistency. Likewise, Stajkovic [40] envisaged that high confidence in one’s capabilities set forth the feeling that one is able to achieve the goals, which enables oneself to perform the pertinent actions. Therefore, self-efficacy leads to changes in the personal initiatives of individuals. The spirit of self-efficacy among employees converts actions into positive initiatives without significant delay.

Furthermore, challenging goals, set by employees with self-efficacy, gives them the confidence to be successful workers [41]. Multiple studies supported the influence created by self-efficacy on employees’ behaviors [8,41]. However, indications that self-efficacy can be used as a mediator in the ethical leadership–employee behavior relationship are at relatively nascent stages. Walumbwa et al. [42], however, proposed that self-efficacy may serve as a mediator between ethical leadership–employee performance relationships. He grounded the SLT (social learning theory) of Bandura [9] to argue the reasons for ethical leadership’s effects on performance through self-efficacy. The SLT theory posits that leaders are an example for followers, especially when they are ethical and credible. They are role models through whom followers learn tasks and knowledge, which evokes an impact on their behaviors. Therefore, as proposed by Walumbwa et al. [42], the current study also predicts the mediation effect of self-efficacy between ethical leadership and employee engagement relationship.

**Hypothesis** **4** **(H4).** *Self-efficacy mediates the relationship between ethical leadership and employee engagement.*

### 2.5. Employee Organizational Commitment as a Mediator between Ethical Leadership and Employee Engagement

“Corporate ethical values may also boost employees’ commitment to the organization” [43]. Individuals might perceive a stronger attachment to companies that adopt ethical values [44]. Hunt et al. [15] found that marketing professionals’ commitment was positively related to corporate concern for ethics. Fritz et al. [45] found that organizations could augment individual commitment through ethical compliance and rewards. Leadership behavior changes the level of commitment in followers; hence, commitment becomes a mediator between leadership styles and employee behaviors [46]. Therefore, as proposed in some earlier studies [46,47], the current study also expects organizational commitment to act as a mediator for the relationship between ethical leadership and employee engagement.

**Hypothesis** **5** **(H5).** *Organizational commitment mediates the relationship between ethical leadership and employee engagement.*

### 2.6. Self-Efficacy and Employee Commitment as Mediators

Self-efficacy [48] and employee commitment [47] both intervene in the relationship between ethical leadership and employee engagement. Employee engagement is realized when employees possess autonomy, commitment, and involvement in their work, as these cater for the establishment of a stronger relationship with their organization [19]. Meanwhile, the presence of an ethical leader facilitates employees to feel motivated, energetic, and committed to their work [49]. Macey et al. [36] acknowledged that employees’ engagement enhances when they are given freedom and capacity. In this vein, it is nevertheless assumed that receiving situational cues from the workplace plays a significant role in the enhancement of employees’ motivation [50]. Bellingham [51] argued that the ethical behavior of leaders is targeted towards followers’ development by enabling them to meet challenges that increase their commitment. Hence, this establishes a reason for freedom and confidence to be bestowed upon employees, as they creates commitment in them and make them more engaged towards their work. Thus, the above discussion leads to the following hypothesis:

**Hypothesis** **6** **(H6).** *Self-efficacy and organizational commitment sequentially mediate the relationship between ethical leadership and employee engagement.*

## 3. Materials and Methods

### 3.1. Sample and Procedure

For conducting empirical analysis, the data to be used were collected through self-administered questionnaires from different organizations of Lahore, including banks, multinationals, and universities, etc. The participants were assured regarding the confidentiality of the survey.

A three-wave, time-lagged design of study was opted for in the current research to remove common method biases [52]. Ethical leadership was measured at Time 1 (T1). After completing measurements at T1, a 15-day gap was given, and then measures of self-efficacy and organizational commitment were surveyed at Time 2 (T2). At Time 3 (2 weeks after T2), measures of employee engagement were collected. For every organization, identical procedures were opted for in the collection of data at TI, T2, and T3.

Initially, at T1, 400 participants were given questionnaires. With a response rate of 75%, 300 questionnaires were received as complete and usable. At T2, the distribution of 300 questionnaires was performed to the same participants who responded at T1. Responses of 270 participants were gathered, and 25 questionnaires remained incomplete with some missing information, reducing the usable questionnaires to 245. The response rate at T2 was 82%. At T3, 245 questionnaires were given to the same participants, out of which, 225 questionnaires received responses. However, there were only 211 usable questionnaires with complete information. The response rate at T3 was 86%. Thus, 211 questionnaires were used for the study. The respondents consisted of 112 (53%) male and 99 (47%) females, with the age range of 20–56 years (mean = 32.77 SD = 7.82 years); the marital status showed that 47% were single, whereas 50% of the respondents were married and the remaining 3% were divorced or widowed. The range of education was 14–20 years, with an average level of 16.42 years, and an SD = 2.714 years; 17% were bachelor’s degree holders, 66% were master’s degree holders, and doctorate level participants made up 9% of the total sample.

### 3.2. Measures

#### 3.2.1. Ethical Leadership (EL)

We used a 10-item scale developed by Brown et al. [4] to measure ethical leadership. An example item is “My supervisor defines success not just by results but also by the way that they are obtained”. The scale was scored on a 5-point Likert-type scale ranging from 1 = Almost Never to 5 = Very Often. Cronbach’s alpha for this scale stood at 0.87**.**

#### 3.2.2. Self-Efficacy (SE)

We used an 8-item scale [11] to measure self-efficacy. An example item is “When facing difficult tasks, I am certain that I will accomplish them”. The scale was scored on a 5-point Likert-type scale ranging from 1 = Almost Never to 5 = Very Often. Cronbach’s alpha for this scale resided at 0.87.

#### 3.2.3. Organizational Commitment (OC)

We used a 6-item scale of organizational commitment formulated by Fry et al. [53]. An example of the items in the scale is “I would be very happy to spend the rest of my career with this organization.” The scale was scored on a 5-point Likert-type scale ranging from 1 = Strongly Disagree to 5 = Strongly Agree. For this scale, Cronbach’s alpha was recorded at 0.78.

#### 3.2.4. Employee Engagement (EE)

For the measurement of employee engagement, we used the Utrecht Work Engagement Scale (UWES) by Schaufeli et al. [54]. The scale had 17 items, including “At my work, I feel bursting with energy”. On a 5-point Likert-type scale ranging from 1 = Almost Never to 5 = Very Often, the responses were obtained. Cronbach alpha resided at 0.91, showing the good reliability of the scale.

#### 3.2.5. Control Variables

Prior research conducted by Schaufeli et al. [54] proposed that age, gender, tenure and education can affect employees’ engagement and commitment. Age, gender, and tenure are all related to the dependent variable of the present study, i.e., employee engagement. Therefore, we controlled these variables in this research. As there is an increase in age and tenure, the association with the organization also grows. Moreover, gender also plays its part in employee engagement, as male employees are generally more engaged. Thus, we included these variables in the control variables.

## 4. Results

We adhered to the earlier approaches adopted by researchers for the conduction of data analysis [55]. Primarily, confirmatory factor analysis was performed using AMOS software version 24 for the five-factor measurement model and hypotheses testing. Following confirmatory factor analysis, hypotheses were tested using PROCESS macro analysis. The selection of PROCESS macro analysis was opted for as this approach is regarded as rigorous for the detection of the significance of conditional indirect effects [55].

A sequence of CFA was run to assess the discriminant validity of constructs using AMOS 24, reported in Table 1. The hypothesized four-factor model reveals a good fit (χ^2^/df = 2.41, TLI = 0.90, CFI = 0.93, RMSEA = 0.08). As an alternative three-factor model, the combination of ethical leadership and self-efficacy was also performed. This three-factor model (χ^2^/df = 4.07, RMSEA = 0.12, TLI = 0.48, CFI = 0.50) fits the data significantly worse than the four-factor model. Additionally, the two-factor model and one-factor model were tested, which also showed significantly worse results. The discriminant validity of the studied measures, hence, is supported by these results.

Moreover, Table 2 reveals a bivariate correlation among ethical leadership (EL), self-efficacy (SE), organizational commitment (OC) and employee engagement (EE). The prerequisite of taking SE as a mediator is satisfied, as ethical leadership shows a significant relationship with self-efficacy, SE (r = 0.16, *p* < 0.05). Furthermore, the results show an indication of the possibility of a mediation chain when SE and OC are taken as mediators. The correlation suggests that ethical leadership is related to organizational commitment (r = 0.41, *p* < 0.01) and self-efficacy is related to organizational commitment as well (r = 0.40, *p* < 0.01). A significant positive relationship of all three variables with employee engagement also exists with ethical leadership (r = 0.14, *p* < 0.05), self-efficacy (r = 0.64, *p* < 0.01), and organizational commitment (r = 0.44, *p* < 0.01), as exhibited in Table 2.

### 4.1. Internal Consistency

To evaluate the composite reliability and Cronbach’s alpha of the measurement model, internal consistency was checked [55]. For both the tests of reliability, the value needs to be 0.70 or higher. The reliability coefficient of ethical leadership, self-efficacy organizational commitment and employee engagement is 0.87, 0.87, 0.78, and 0.91, respectively, hence establishing internal consistency for the measurement model.

### 4.2. Convergent and Discriminant Validity

Convergent validity refers to the extent to which two measures that are theoretically related are related empirically as well [56]. For the establishment of the convergent validity, the AVE (Average Variance Extracted) and outer loadings were examined [56], and results are demonstrated in Table 3. The research proposes the criteria that the majority of the indicators should have an outer loading higher than 0.7. Discriminant validity refers to a divergent validity that investigates the extent to which a construct is different from others. The Fornell–Larcker [57] approach and item’s cross-loadings assist this assessment. Adopting this approach, discriminant and convergent validities are showed at a composite reliability of >0.7 and an average variance extracted of >0.5, as revealed in Table 3; hence, convergent and discriminant validity is proven.

### 4.3. Multicollinearity

Due to the presence of multiple mediators, the estimation of tolerance test and VIF (variance inflation factor) was performed to check the assumptions of multicollinearity. The results of multicollinearity are exhibited in Table 4. For all variables used in the study, VIF < 10 reveals that the data have no multicollinearity. Moreover, the Durbin–Watson value of the data shows no autocorrelation as it stands at 1.23, which is well within the acceptable range between 0–4. The Durbin–Watson value of our data indicates a positive sequential correlation that allows us to proceed with regression analysis.

### 4.4. Testing of Hypotheses

For the testing of hypotheses, sequential mediation was performed. We used Hayes’ PROCESS [58], which is considered one of the best approaches to handle mediation [56]. To examine the theory with parameter estimates, we applied Hayes’ PROCESS model 6. PROCESS procedures were applied to investigate the significance of indirect effects. To minimize the drawbacks of a small sample size, a 2000 re-sample BC bootstrapping method was employed [58]. The results of the mediation model are shown in Table 5.

**Figure 1:**
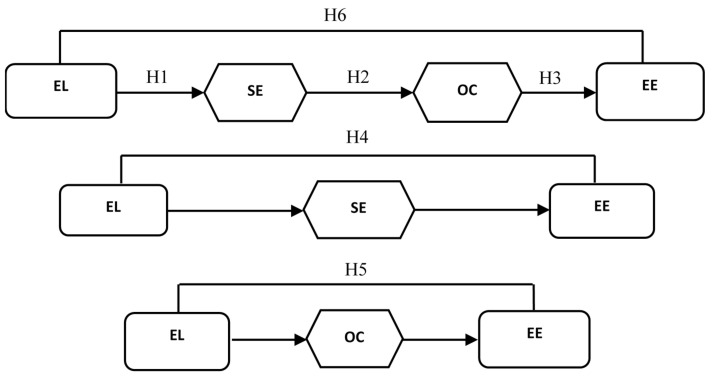
Sequential Mediation Model. EL: ethical leadership; SE: self-efficacy; OC: organizational commitment; EE: employee engagement.

The path analysis of our sequential mediation model (Figure 1) reveals the direct and indirect effects of mediated pathways. According to Preacher and Hayes [58], with the 2000 re-sample bootstrapping method, 90% CI of the direct and indirect effects were determined. The results of the sequential mediation model revealed that the direct effect remained statistically non-significant CI_90% confidence level_ [−0.135, 0.057] concerning the mediating effects of self-efficacy and organizational commitment on the relationship between ethical leadership and work engagement, whereas the corresponding indirect effect between ethical leadership and employee engagement via the sequential mediation of self-efficacy and organizational commitment was found to be statistically significant CI_90% confidence level_ [0.070, 0.272]. As the indirect effect remained significant while the direct effect showed non-significant results, we established that self-efficacy and work commitment fully mediated the ethical leadership–employee engagement relationship.

Table 5 also reveals the total effect of the model, including the direct effect of ethical leadership on employee engagement. The results reveal the significance of the total effect by showing that the upper and lower limits [0.006, 0.240] do not contain a zero value as both limits are positive. Considering of the mediating effect of self-efficacy on ethical leadership–employee engagement relation, the findings depicted positive association of ethical leadership (EE) and self-efficacy (SE) with β = 0.147, *p* < 0.05, CI_90% confidence level_ [0.022, 0.273]. Moreover, the LLCI and ULCI are positive, not having a zero value. Hence, it supports Hypothesis 1. The prediction of the second hypothesis stated that the SE is positively related to organizational commitment. The results revealed that β = 0.357, *p* < 0.001, CI_90% confidence level_ [0.234, 0.481], hence supporting Hypothesis 2. For the third hypothesis, the study stated that organizational commitment is positively related to employee engagement. The regression coefficient of the results depicted is β = 0.216, *p* < 0.001, CI_90% confidence level_ [0.075, 0.340]; hence, it supports Hypothesis 3 of the current research.

The mediation of self-efficacy between ethical leadership and employee engagement is significant with CI_90% confidence level_ [0.016, 0.144], as shown in the first indirect path, supporting Hypothesis 4. In the third indirect path, the mediation of organizational commitment between ethical leadership and employee engagement shows CI_90% confidence level_ [0.022, 0.146], hence supporting our Hypothesis 5. The sequential mediation of self-efficacy and organizational commitment between ethical leadership and employee engagement is shown in the second indirect path, and reveals CI_90% confidence level_ [0.002, 0.031], supporting Hypothesis 6 as predicted in our study. The results of all indirect paths are significant, as zero is not included in their upper and lower limits. Moreover, the total direct effect without mediators was statistically insignificant; hence, the study results revealed that self-efficacy and organizational commitment fully mediate the relationship between ethical leadership and employee engagement.

## 5. Discussion

In this study, we conceptualized and examined self-efficacy and employee commitment as mediators between ethical leadership and employee engagement. The results revealed that the relationship between ethical leadership and employee engagement is significantly mediated by self-efficacy and employee commitment. Further, the relationship between ethical leadership and employee engagement was fully mediated by self-efficacy and organizational commitment. The data analysis result reveals the support of all six hypotheses. The prediction of the first hypothesis regarding a positive relationship between ethical leadership and employees’ self-efficacy is supported. Earlier studies also support the positive relationship between an ethical leader and an employee’s self-efficacy behavior [59]. The prediction of the second hypothesis that self-efficacy has a positive impact on employees’ commitment is also supported by our results, as well as previous literature [43,60,61]. The third hypothesis is also supported; the results reveal that organizational commitment is positively related to employee engagement, as contended in earlier studies [62]. The mediation of self-efficacy is significantly substantiated in Hypothesis 4. The mediation of organizational commitment is also established in Hypothesis 5. The sequential mediation of self-efficacy and organizational commitment is established in Hypothesis 6, as predicted in our study.

In line with the hypotheses, the findings suggest that positive behavior of leadership by showing ethical conduct influences employee engagement. When leaders are credible, they are role models for their followers from whom they seek guidance for their tasks. This learning experience increases their belief in their abilities that enhance their confidence to be successful in meeting emerging challenges. The credibility and support of ethical leaders augment followers’ learning and confidence, hence increasing their self-efficacy. Walumbwa et al. [48] argued that self-efficacy plays a significant part in this relationship. According to Zhou, Wang, Chen, and Shi [28], leaders’ conduct and resources in the organization leave a greater impact on followers’ behaviors. Furthermore, the tenets of the conservation of resource theory contend that resources in the workplace are invested to build new resources [25]. Ethical leaders provide resources whether material or socioemotional, hence allowing followers to reciprocate by investing these valued resources to build more resources, as per expectations. The positivity in the environment created by leadership enhances commitment in employees with their organizations [32]. This commitment to their organizations further leads to the development of engagement among them. According to Aquino and Reed [63], psychological determinants and situational circumstances both determine behavior, hence providing an insight into the importance of a leader’s conduct and establishing an environment that fosters self-efficacy and commitment in followers as a catalyst for employees’ engagement.

## 6. Theoretical and Practical Implication

The findings of the current study generated some interesting implications for theory and practice. First, a scarcity of research amid the understudy variables generated the necessity to empirically test and formulate a theoretical model that explores the direct and indirect association between the aforementioned study variables. The results empirically advocate that ethical leadership influences self-efficacy and enhances organizational commitment, which in turn leads to employee engagement. The study also ascertains the sequential mediation models of three pathways. According to our literature review, this study is the first to ascertain the sequential mediating effect of self-efficacy and organizational commitment in the extant literature of ethical leadership and employee engagement relations. The outcomes of the sequential mediation of self-efficacy and organizational commitment to ethical leadership–employee engagement relation revealed complete mediation. To the best of our knowledge, a study creating a link for all the variables in the current study is not found in the literature. Hence, the findings have contributed as an addition to the existing body of knowledge of ethical leadership–employee engagement relations.

The study also identifies how ethical leadership facilitates the work engagement of followers, thus addressing the research gap mentioned by Carasco et al. [23]. Moreover, it adds to the research on the direct link between an ethical leader’s attributes and their follower’s engagement at the workplace, as well as the underlying mechanism that facilitates this link, addressing another future research area [1,24].

The empirical findings of the theoretical model suggested its significant implications for followers. The study analyses results, with respect to the conservation of resource theory, propose to leaders that their positive ethical leadership style works as a great organizational resource, which encourages employees to invest their resources in enhancing their engagement level. This increases energy levels for employees and enhances their efficacy.

Further, the study seeks to draw the attention of employers to inculcate the behavior of ethical leadership in their leaders through counselling and training, as this will enable organizations to develop such an organizational culture where employee development and encouragement is the foremost concern of the leaders. This might help organizations to have a committed and engaged workforce with more productivity and less turnover in today’s competitive arena.

## 7. Limitations and Future Research

Despite offering theoretical, practical and empirical contributions, as most of the research, our study is not free of limitations. First, the sample of the study is relatively small. The employment of the 2000 re-sample bootstrapping method has minimized this limitation and provided room for relative generalization; however, while generalizing the results of this study, caution needs to be exercised. A second limitation concerns the completion of the study’s variables via self-reports; this type of datum has several well-known shortcomings [52]. Though individuals provide a better assessment of themselves and their perceptions, sometimes, relatively unintentional or insincere responses by the respondents affect the study results. This limitation needs to be kept in mind while generalizing the results. In the present study, we opted for a three-wave time-lagged study design, yet future studies may employ multi-sources for the collection of data and may opt for a longitudinal study design to avoid common method biases.

Future research may examine some consequences of employee engagement, in addition to the various antecedents examined in research so far: for instance, the impact of employee engagement on life satisfaction. Moreover, future research may include measures of workplace spirituality. It is suggested that employee engagement is good for individuals intrinsically as well as for the organization for its productive outcomes [64,65]. However, as identified by Shuck et al., [66], there are more refined sub-dimensions of engagement, such as emotional, cognitive, and behavioral engagement, etc. These sub-dimensions may be accounted for in future research.

## 8. Conclusions

The study offers multiple considerable contributions to theory and research. We demonstrated that ethical leadership influences employees’ engagement when self-efficacy and organizational commitment sequentially mediate the relationship. Earlier studies used self-efficacy as a moderator; however, this study focused on the aspects of self-efficacy that are enhanced by ethical leadership. The results show that the entire variance that explains ethical leadership on employee engagement is possible with the effects of self-efficacy and organizational commitment. To put it simply, for achieving ethical leadership–employee engagement relation, the plausible means are employees’ self-efficacy and organizational commitment. Thus, this research offers strategies to leaders to foster a culture that bridges self-efficacy to organizational commitment so that employees may be engaged with their work.

## Figures and Tables

**Table 1:** Results of CFA—confirmatory factor analysis.

Factor	χ^2^/df	RMSEA	GFI	TLI	CFI
4 factor model	2.41	0.08	0.92	0.90	0.93
3 factor model *	4.07	0.12	0.49	0.48	0.50
2 factor model **	4.28	0.13	0.47	0.44	0.47
1 factor model	4.76	0.13	0.42	0.40	0.42

Note: *n* = 211. * Three-factor model: EL and SE were combined; ** two-factor model: OC, EL and SE were combined; χ^2^ = chi-square; df = degree of freedom; GFI = goodness of fit; CFI = comparative fit index; TLI = Tucker–Lewis Index; RMSEA = root mean square error of approximation.

**Table 2:** Matrix of correlations.

Variables		Mean	SD	1	2	3	4	5	6	7
1.	Age	32.70	7.78							
2.	Tenure	5.92	4.99	0.75 **						
3.	Education	16.72	1.41	0.18 **	0.11					
4.	EL	3.67	0.68	−0.16 *	−0.10	0.05	***(0.87)***			
5.	SE	3.94	0.63	0.08	0.09	−0.02	0.16 *	***(0.87)***		
6.	OC	3.77	0.66	−0.04	0.06	0.24 **	0.41 **	0.40 **	***(0.78)***	
7.	EE	3.82	0.58	0.06	0.05	0.012	0.14 *	0.64 **	0.44 **	***(0.91)***

Note: *n* = 211, ** *p* < 0.01 (two-tailed), * *p* < 0.05 (two-tailed). Cronbach’s alpha is shown in the parentheses.

**Table 3:** Convergent and discriminant validity.

	CR	AVE	MSV	ASV	EL	SE	OC	EE
EL	0.838	0.510	0.128	0.842	**0.714**			
SE	0.895	0.522	0.006	0.919	0.076	**0.722**		
OC	0.743	0.505	0.128	0.802	0.358	0.719	**0.710**	
EE	0.922	0.545	0.001	0.936	0.023	0.713	0.648	**0.738**

CR = composite reliability; AVE = average variance extracted; MSV = maximum shared variance; ASV = average shared variance.

**Table 4:** Multi-collinearity and autocorrelation diagnostic.

Variables	Collinearity Statistics	
	Tolerance	VIF *
OC	0.720	1.388
EE	0.833	1.201
SE	0.843	1.186
*Durbin–Watson statistic 1.234*		

VIF * = variance inflation factor.

**Table 5:** Path coefficients and indirect effects of mediation models.

	Path Coefficients			Indirect Effects	
	SE	OC	EE	Boot LLCI	Boot ULCI
From → To					
EL	0.147 *	0.348 ***	−0.309		
SE		0.357 ***	0.509 ***		
OC			0.216 ***		
Total Indirect Effects				0.070	0.272
Indirect Effect					
EL → SE → EE				0.016	0.144
EL → SE → OC → EE				0.002	0.031
EL → OC → EE				0.022	0.146
Direct Effect					
EL → EE				−0.135	0.057
Total Effect					
EL → EE				0.006	0.240

*** *p* < 0.001, * *p* < 0.01; LLCI = Lower Limit Confidence Interval; ULCI = Upper Limit Confidence Interval.

## Data Availability

The datasets generated and analyzed in the current study are available from the corresponding author upon reasonable request.
